# Deep Frying in Extra-Virgin Olive Oil: Evaluating the Influence of the Type of Food and Breading on the Degradation of Phenolic Compounds

**DOI:** 10.1021/acs.jafc.6c00555

**Published:** 2026-05-27

**Authors:** Ana Castillo-Luna, Feliciano Priego-Capote

**Affiliations:** † Department of Analytical Chemistry, 16735 University of Córdoba, Campus of Rabanales, Córdoba 14014, Spain; ‡ Chemical Institute for Energy and Environment (IQUEMA), University of Córdoba, Campus of Rabanales, Córdoba 14014, Spain; § Maimónides Institute of Biomedical Research (IMIBIC), Reina Sofía University Hospital, University of Córdoba, Córdoba 14014, Spain; ∥ Consortium for Biomedical Research in Frailty & Healthy Ageing CIBERFES, Carlos III Institute of Health, Madrid 28029, Spain

**Keywords:** deep-frying, nonbreaded foods, breaded food, extra virgin olive oil, phenolic compounds, LC−MS/MS

## Abstract

Deep-frying in vegetable oils is a widespread practice around the world. The use of extra virgin olive oil (EVOO) for frying is supported by the presence of bioactive compounds such as phenols, which contribute to oxidative stability and are partially transferred to the fried food. We monitored by LC–MS/MS, the degradation of phenols in Picual and Arbequina EVOOs during consecutive frying cycles of different types of foods. The phenolic content decreased significantly (p-value <0.0001) in oils, but the decrease was more pronounced with nonbreaded foods (around 93% for chicken wings and anchovies) than with breaded foods, with a degradation between 70% and 78%. An exception was found for potatoes that behaved similarly to breaded foods, and this was attributed to their composition. Comparative analysis of the EVOOs revealed that secoiridoids primarily respond to oxidation during frying, but the aglycone isomers have a stronger protective capability than oleacein and oleocanthal.

## Graphical abstract

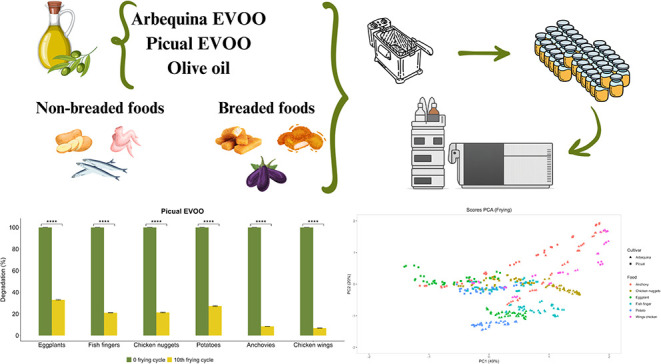

## 1 Introduction

Virgin olive oil (VOO) is considered the main fat source in the Mediterranean diet due to the health benefits attributed to its chemical composition.^1^ VOO consists of two main fractions: the saponifiable fraction (around 98% of the total weight composition) is composed of acyl glycerides, phospholipids, and sterol esters, among others, while the unsaponifiable fraction (2% of the total content) is formed by a heterogeneous group of chemical compounds such as phenols, terpenes, volatiles, hydrocarbons, or tocopherols.^2^ Among them, it is worth highlighting the phenolic compounds because of their specific features. First, the European Food Safety Authority (EFSA) established a health claim in the European Regulation EU 432/2012 for olive oils that provide at least 5 mg of hydroxytyrosol and its derivatives in a daily intake of 20 g. The claim is related to positive effects on the protection of blood lipids against oxidative stress.^3^ Second, phenols are also associated with the organoleptic properties of VOOs. Pedan, Popp, Rohn, Nyfeler, and Bongartz (2019) studied the relation between phenols, cultivars, and organoleptic characteristics, and they showed that these compounds play a relevant role in some distinctive positive attributes of VOO.^4^ According to Barbieri, Bendini, Valli, and Toschi (2015), the bitterness and pungency perceptions are related to the phenolic composition of VOO.^5^ Finally, phenols play a key role in the oxidative stability of VOOs due to their antioxidant capability, which depends on the phenolic relative profile and not on the total phenol content.^6^

The individual and total phenolic content in VOOs depends on multiple factors that encompass the cultivar, agronomic practices, growing techniques, processing, packaging, and storage.^7^ Daskalaki, Kefi, Kotsiou and Tasioula-Margari (2009) evaluated the degradation of phenols during the heating and storage of VOO. They observed that the degradation of these compounds depended on the heating temperature. They heated VOO at 180 and 100 °C and verified that the higher the temperature, the greater the degradation degree. Moreover, they studied the degradation of phenols during the storage and concluded that the phenolic decrease was influenced by the unsaturation of olive oil, headspace oxygen availability, and temperature.^8^ Castillo-Luna, Criado-Navarro, Ledesma-Escobar, López-Bascón and Priego-Capote (2021) stated that the total and individual phenolic content of extra virgin olive oils (EVOOs) was modified during storage for one year even if it was kept under optimal conditions.^9^

Nowadays, several cooking techniques use vegetable oils for food processing. Ambra, Lucchetti and Pastore (2022) reviewed these techniques, such as deep-frying, pan-frying, roasting, air-frying, microwaving, and boiling.^10^ Among them, frying is one of the most widely used cooking techniques for processing different types of foods, which are immersed in oil at 180 °C during a specific time. Currently, numerous studies suggest that VOO is the best oil for deep-frying due to its lipidic composition, enriched with monounsaturated fatty acids, and the phenolic presence with a protective effect during cooking.^11–13^ Nevertheless, deep-frying affects the total phenolic content in absolute and relative terms.^14–18^

Despite this evidence, the influence of other factors involved in frying such as the food composition or the breading in the degradation of phenolic compounds has not been analyzed. The hypothesis is that the frying process could modify the phenolic profile of the oils depending on the type of food (fish, meat, or vegetables) and the processing of breaded or nonbreaded foods. With these premises, this research plans the next aims: (i) to study the behavior of phenolic compounds during ten frying cycles by comparing two EVOOs, Arbequina and Picual, with different phenolic profiles; (ii) to evaluate the influence of the type of food on the degradation of phenols; and (iii) to study the effect of breading on the degradation kinetics of individual phenols.

## 2 Materials and Methods

### 2.1 Chemicals and Reagents

Deionized water (18 MΩ·cm) from a Millipore Milli-Q water purification system (Merck Millipore, Bedford, MA, USA) and mass spectrometry (MS) grade methanol (MeOH) from Scharlab (Barcelona, Spain) were employed for the preparation of samples and chromatographic mobile phases. LC/MS formic acid from Fisher Scientific (Hampton, NH, USA) was used as an ionizing agent.

Hydroxytyrosol and tyrosol were purchased from Extrasynthese (Genay, France), the secoiridoid derivatives oleacein (3,4-DHPEA-EDA) and oleocanthal (p-HPEA-EDA) were acquired from Phytolab (Vestenbergsgreuth, Germany), and oleuropein and ligstroside aglycones from TRC (Ontario, Canada, Greece). Syringaldehyde from Sigma-Aldrich (St. Louis, MO, USA) was used as an internal standard (IS) to control the LC–MS/MS performance during the analysis of all samples.

### 2.2 Sample Preparation

Two monocultivar EVOOs, “Picual” and “Arbequina”, produced in 2022, were provided by an organic producer (Oro del Desierto, Almería, Spain). These two EVOOs were selected for their extraordinary quality and their high phenolic content. Furthermore, Picual and Arbequina EVOOs possess different phenolic profiles. Picual EVOOs are highlighted by their high content in oleuropein and ligstroside aglycone isomers, while Arbequina EVOOs are enriched with oleocanthal and oleacein. Ordinary olive oil (OO) was purchased from a conventional supermarket. Due to its low phenolic content, this olive oil was included as a reference control to evaluate the role of EVOO phenols on the oxidative stability.

Different food products were also acquired in a local supermarket. These were potatoes and eggplants (vegetables), chicken wings and chicken nuggets (meat), and anchovies and hake fish fingers (fish). All foods were diced into 2 cm^3^ pieces, except for the eggplants, which were cut into 1 cm × 7 cm strips. Eggplant strips, chicken nuggets, and hake fish fingers were prepared by breading them with egg and flour.

Each food was fried independently in 1 L of each type of oil (Picual, Arbequina, and olive oil) for ten cycles at a temperature of 180 °C, in an enclosed domestic deep-fryer (37680 model, San Ignacio Kitchen, Spain), with an oil-air contact surface area of 225 cm^2^. The thermostat controlled the temperature during each cycle. The oil was preheated for approximately 5 min until it reached 180 °C before the first cycle. Each frying cycle was defined by the food batch (20 g, approximately 4–6 pieces) permanence time in the oil, which was around 2.5 min. To minimize oil loss, the food was drained over the deep-fryer before removal. Posteriorly, a 3 mL aliquot of the oil was collected immediately after each batch was removed. The next cycle started once the temperature stabilized. The total oil volume loss at the end of the ten cycles was considered negligible, at around 4%. All samples were stored at −80 °C until analysis.

Phenolic compounds were analyzed according to the protocol described by Miho et al. (2018). 250 μL of *n*-hexane was added to 0.5 g of oil and vortexed for 30 s. Afterward, 2 mL of 80:20 (v/v) MeOH:H_2_O with the IS (1 mg/L) were added for liquid–liquid extraction. The hydroalcoholic phase was separated by centrifugation at 900 × *g* for 8 min and isolated for analysis. This research involved a total number of 183 samples involving 6 foods × 3 oils × 10 frying cycles + 3 initial oils. Three replicates were analyzed for each aliquot.^19^

### 2.3 LC–MS/MS Analysis

Phenolic analysis was carried out with an Agilent 1200 series LC system (Palo Alto, CA, USA) in negative mode with electrospray ionization (ESI) coupled to an Agilent 6410 triple quadrupole (QqQ) tandem mass spectrometer. A Mediterranea C_18_ (3 μm particle size, 5.0 × 0.46 cm i.d.) chromatographic column from Teknokroma (Barcelona, Spain) was utilized for the separation of phenolic compounds. A Mediterranea C_18_ precolumn (4 μm particle size, 5.0 × 0.30 cm i.d.) was used to protect the analytical column. The temperature of the chromatographic column was set at 30 °C. Deionized water and methanol were used as mobile phases (phase A and B, respectively), both with 0.1% (v/v) formic acid as the ionizing agent. The chromatographic gradient began with 50% B for 0.5 min, followed by an increase to 80% B from 0.5 to 2 min. Then, from 2 to 5 min, it increased to 100% B, which was maintained until 6.5 min to ensure the correct elution of analytes. Finally, the initial conditions were restored from 6.5 to 10.5 min, followed by 1.5 min postrun for re-equilibration of the column. The injection volume for EVOO and olive oil extract samples was 5 μL, and the flow rate was 0.4 mL/min.

For the screening of phenolic compounds, a multiple reaction monitoring (MRM) method developed by Miho et al. (2018) was used (Supplementary Table S1). The ESI unit operated in negative ionization mode with a nebulizer pressure of 50 psi, capillary voltage of 3000 V, temperature of 300 °C, and drying gas (N_2_) flow rate of 10 L/min.^19^

### 2.4 Quantitative Analysis of Phenols

Refined sunflower oil spiked with a multistandard phenolic solution at different concentrations (1 to 20 mg/kg) was used to develop calibration models. These spiked aliquots of sunflower oil were processed by liquid–liquid extraction with 2 mL of 80:20 MeOH:H_2_O solution. Each extract was analyzed by LC–MS/MS to obtain the calibration models. We used the ratio between the peak area of each phenolic compound and that of the IS (Supplementary Table S2). Oleokoronal and oleomissional were quantified using the calibration models developed with the monoaldehyde closed isomers (ligstroside aglycone and oleuropein aglycone, respectively). Similarly, oleocanthalic acid was quantified using the calibration model of oleocanthal.

### 2.5 Analysis of the Oxidative Stability of Crude Oils and Oils Subjected to Frying

The oxidative stability of the oils was analyzed using an Oxitest Oxidation Test Reactor (Velp Scientifica Srl, Usmate, Italy) by applying the AOCS Cd-12c-16 official method.^20^ Thus, 0.5 g of each oil (crude oils and those sampled after frying foods) was oxidized at 110 °C with 7 bar of oxygen pressure. Each oil was analyzed in triplicate. OXISoft software was used to calculate the Induction Period (IP) based on the oxidation curves.

### 2.6 Statistical Analysis

For processing and statistical analysis, we used the free software *R* (version 4.4.1., http://www.r-project.org/). A two-way analysis of variance (ANOVA) and Mauchly’s sphericity test were used to determine statistical significance between frying cycles using the stats package (version 0.1.0, https://CRAN.R-project.org/package=STAT). Kruskal–Wallis test (95% confidence interval) and pairwise comparisons (Wilcox test) were performed to study the significance of the phenolic degradation percentage and oxidative stability curves with the plyr package (version 1.8.9, http://had.co.nz/plyr). Moreover, Principal Component Analysis (PCA) was carried out to identify discrimination patterns between EVOOs and olive oil samples. PCA was realized with the mixOmics package (version 6.24.0, https://CRAN.R-project.org/package=mixOmics) by setting eight components with scaled and centralized data. Finally, a Hierarchical Cluster Analysis (HCA) was performed, using Ward’s minimum variance method and the Euclidean distance metric. HCA was carried out with the dendextend package (version 1.19.1, https://cran.r-project.org/web/packages/dendextend/index.html).

## 3 Results and Discussion

### 3.1 Phenolic Profile of EVOOs and Olive Oil

The EVOOs and the ordinary olive oil selected for this research were analyzed according to the methodology proposed by Miho et al. (2018) and used in different studies.^9,19,21–23^ Both EVOOs contained an elevated concentration of phenolic compounds, reaching the value established in the health claim for olive oil recognized by the EFSA. The health claim is considered with a daily intake of 20 g of olive oil that should contain at least 5 mg of hydroxytyrosol, tyrosol, and derivatives.^3^ Arbequina EVOO presented 619 mg/kg of total phenols, highlighting the content of oleacein and oleocanthal (479 mg/kg). On the other hand, Picual EVOO presented 814 mg/kg of total phenols, characterized by the high concentration in oleuropein and ligstroside aglycone isomers, 639 mg/kg (Table 1). Thus, the two selected EVOOs reported clear differences in the relative content of phenols according to the *f* and *h* factors proposed by Miho et al. (2021) to represent the phenolic profile of VOOs. Hence, the *f* factors (defined as the ratio of oleuropein and ligstroside aglycone isomers to oleacein and oleocanthal) of “Arbequina” and “Picual” EVOOs were 0.29 and 3.7, respectively, which point out the two different phenolic profiles. Miho et al. (2021) demonstrated that the *f* factor plays a crucial role in the oxidative stability of EVOOs. Concerning the *h* factor (defined as the ratio of hydroxytyrosol conjugated derivatives to tyrosol conjugated derivatives), this parameter was similar in both monocultivar EVOOs because of the similar concentration of hydroxytyrosol and tyrosol conjugated secoiridoids (oleuropein aglycone isomers and oleacein versus ligstroside aglycone isomers and oleocanthal) in each oil.^21^ Therefore, the phenolic profile of both EVOOs was different by the elenolic acid moiety of the secoiridoids model structure (Supplementary Figure S1). Regarding the ordinary olive oil, it contained a low concentration of total phenols (37.5 mg/kg), which is explained since this product contains mainly refined olive oil not reaching the minimum values established by the EFSA.

**Table 1:** Phenolic Concentration Determined in the EVOOs and Ordinary Olive Oil Selected for the Study^a^

	**Concentration** (mg/kg)		
Phenol	Arbequina	Picual	Olive oil
Oleocanthal	171	102	7.1
Oleacein	308	69.7	1.3
Oleuropein aglycone	61.2	167	11.8
Oleomissional	45.6	328	1.3
Ligstroside aglycone	15.0	43.9	8.1
Oleokoronal	14.7	100	4.0
Hydroxytyrosol	2.0	2.0	0.02
Tyrosol	1.6	1.0	3.9
Total phenols	619	814	37.5

a Variability in concentrations was always below 10%, expressed as relative standard deviation.

### 3.2 Influence of Deep-Frying on the Total Phenolic Content

In this research, six different foods were subjected to a frying process at 180 °C as a standardized temperature. These foods were selected according to two different criteria: the type of food and the presence/absence of breading. Thus, vegetables (potatoes and eggplants), fish (anchovies and fish fingers), and meat (chicken wings and nuggets) were used in this experiment. In addition, three of these foods were nonbreaded (potatoes, chicken wings, and anchovies), and the other three were breaded (eggplant fingers, chicken nuggets, and fish fingers). The three olive oils were subjected to ten consecutive deep-frying cycles. Each cycle involved the use of a new portion of food.

Phenolic compounds were quantified only in the experiments involving the two EVOOs due to the reduced content in the olive oil. The quantitative data of the individual phenolic compounds were analyzed by principal component analysis (PCA) to identify the main variables explaining the change in the phenolic profile of olive oils subjected to deep frying (Figure 1). The PCA score plot revealed that the primary source of variability (around 56% for PC1) was the cultivar due to the differentiation between Arbequina and Picual samples based on their specific phenolic profiles. Concerning the type of food, it is worth mentioning that EVOO samples obtained after frying the two nonbreaded foods, chicken wings and anchovies, were discriminated against those used for breaded foods and potatoes (PC2 21%). In fact, EVOO samples used for frying potatoes were in the same cluster as those used for processing breaded foods. The loading plot revealed the discriminatory behavior of the aglycone isomers of oleuropein and ligstroside, representative for Picual EVOO, and that of oleacein and oleocanthal for Arbequina EVOO (Figure 1).

**Figure 1:**
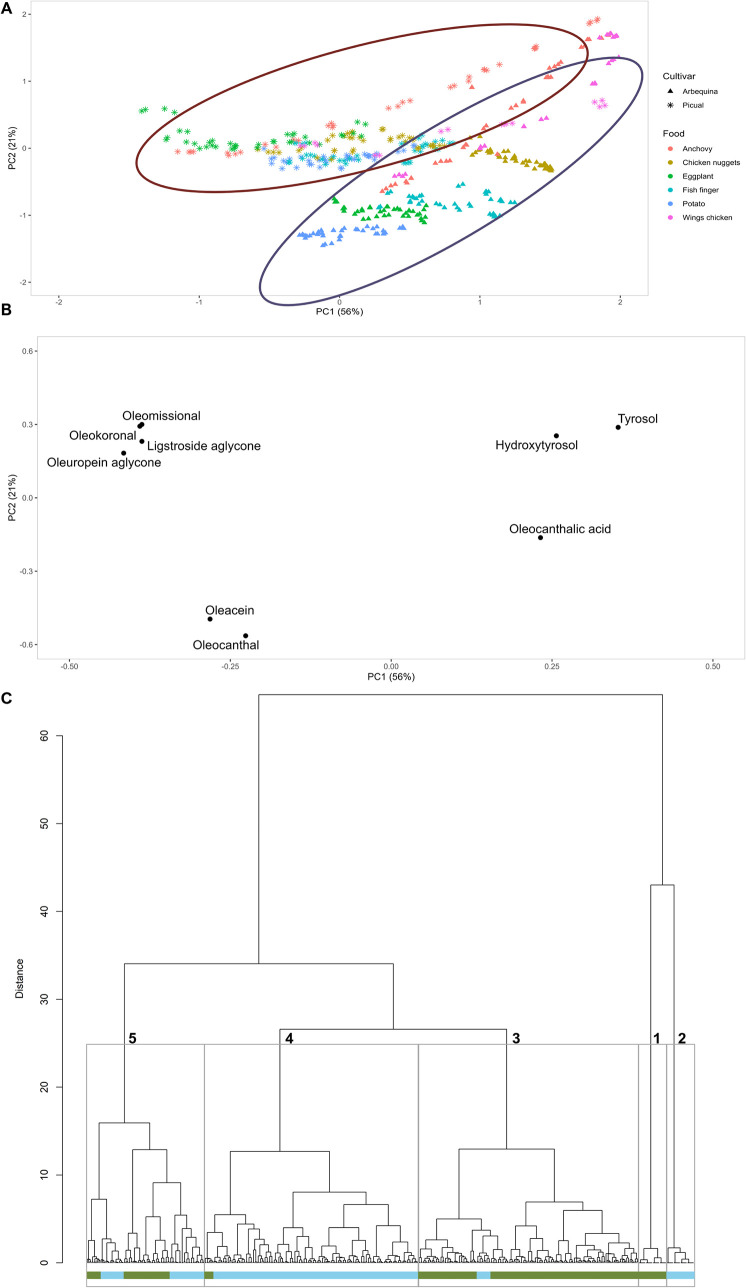
Unsupervised multivariate analysis showing the phenolic variability in olive oil, Arbequina EVOO, and Picual EVOO samples after frying breaded food (eggplants, chicken wings, and anchovies) and nonbreaded foods (potatoes, chicken nuggets, and fish fingers). (A) PCA scores plot; (B) PCA loadings plot; (C) HCA dendrogram plot (green and blue colors for Picual and Arbequina oils, respectively).

To provide statistical rigor to these groupings, a Hierarchical Cluster Analysis (HCA) was performed (Figure 1). The HCA dendrogram, using Ward’s method and the Euclidean distance metric, confirmed the existence of five primary clusters. Clusters 1 and 2 consisted of initial Picual and Arbequina EVOOs, respectively. Clusters 3 and 4 were formed by 93.8% and 95.7% of Picual and Arbequina oils subjected to frying, respectively. Finally, cluster 5 grouped Arbequina and Picual oils sampled after the fifth frying cycle of two nonbreaded foods, particularly, chicken wings and anchovies. This dual multivariate analysis confirms that the dominant factors defining the phenolic evolution during the frying process are the oil type and the cultivar.

The phenolic degradation measured in oils subjected to frying was higher than 60% after the 10th cycle (*p*-value of <0.0001, Figure 2). The highest decrease effect was observed with anchovies (91.5% in Picual and 93.5% in Arbequina) and chicken wings (93.1% in Picual and 93.6% in Arbequina), both corresponding to nonbreaded foods. On the contrary side, the lowest decrease for both EVOOs was found with the two vegetables, eggplants (67.1% in Picual and 66.3% in Arbequina,) and potatoes (72.7% in Picual and 68.3% in Arbequina), which could be explained by the fact that vegetable foods also contain compounds that contribute to oxidative stability, including phenolic compounds. Particularly, previous studies have described the phenolic content of eggplant and potato, which also contributes to oxidative stability during frying and preserves secoiridoids.^11,15,24^ Finally, breaded meat and fish, fish fingers (78.9% in Picual and 76.4% in Arbequina), and chicken nuggets (78.7% in Picual and 78.6% in Arbequina) experienced a lower decrease than nonbreaded fish and meat. During deep-frying, it is well-known that the loss of water from nonbreaded foods is faster than from breaded foods since the breading preserves the moisture content. Therefore, a higher amount of water is released into the medium when frying nonbreaded foods. Since phenols are polar compounds, they are partially transferred to the oil–water interphase, where phenols act against oxidative processes enhanced by temperature. This effect justifies the highest degradation of phenols when frying nonbreaded foods.^11^

**Figure 2:**
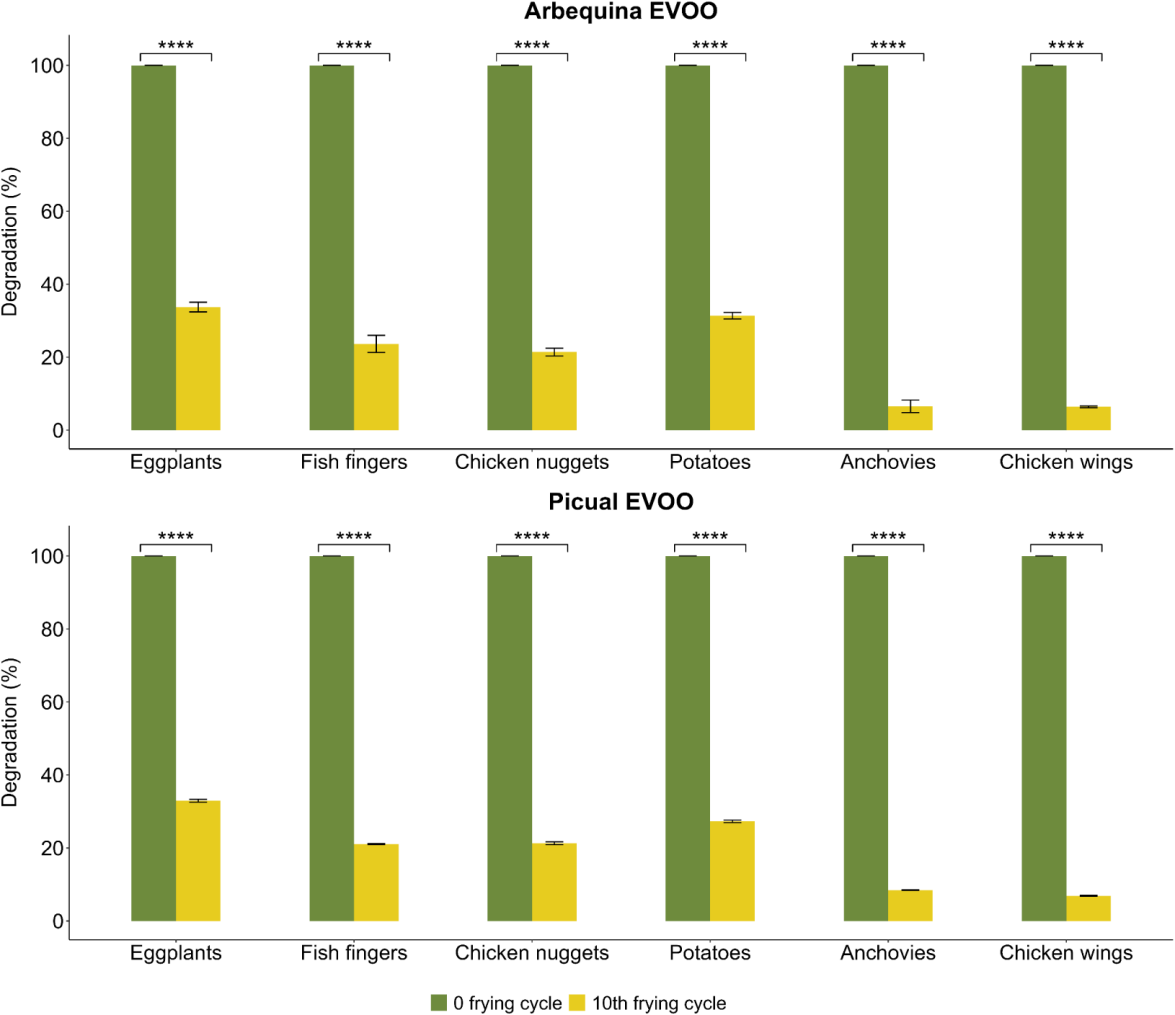
Degradation of the total phenolic content (expressed as %) in olive oil samples after ten cycles of deep-frying. The first three groups represent breaded foods (eggplants, chicken wings, and anchovies), and the last three groups represent nonbreaded foods (potatoes, chicken nuggets, and fish fingers). Significant differences were determined by the successive contrast test and are labeled as “****p-value <0.0001”, “***p-value 0.0001–0.001”, “**p-value: 0.001–0.01”, “*p-value: 0.01–0.05”,and “ns *p*-value >0.05”. Concentrations were normalized before statistical analysis.

Figure 3 shows the decreasing trends observed in the total phenolic content in the different experiments during the frying cycles. Picual and Arbequina EVOOs reported quite similar patterns as different trends can be distinguished between breaded and nonbreaded foods. Thus, the decrease in total phenolic content was faster in nonbreaded foods as compared to breaded foods. In fact, the total phenolic content was reduced by about 80% and 90% in Picual and Arbequina EVOOs, respectively, after the fifth frying cycle when anchovies and chicken wings were processed. In the same vein, the decrease in total phenolic content in breaded foods after the 10th frying cycle was 80% and 75% in Picual and Arbequina EVOOs, respectively, for fish fingers and chicken nuggets. A particular case was found for the two vegetables, potatoes and breaded eggplants, since the phenolic degradation after the 10th cycle in both foods was only 65% and 70% in Picual and Arbequina EVOOs, respectively.

**Figure 3:**
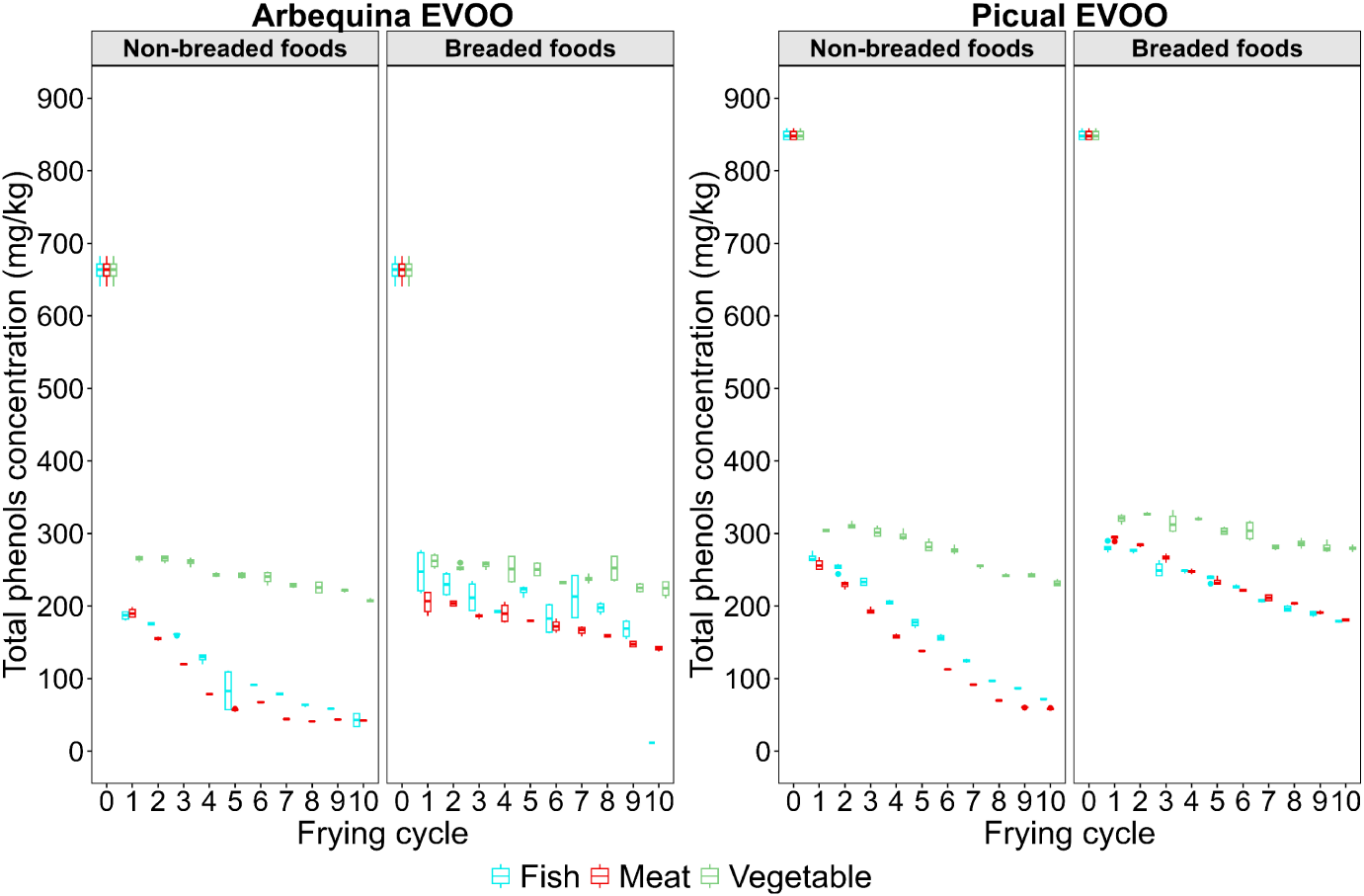
Progressive degradation of the total phenolic content in Arbequina and Picual oils during ten frying cycles of nonbreaded foods (red, chicken wings; blue, anchovies; green, potatoes) and breaded foods (red, chicken nuggets; blue, fish fingers; green, eggplants). Concentrations were normalized before statistical analysis.

### 3.3 Study of the Evolution of Individual Phenols during Deep-Frying

The most abundant phenols found in olive oil were quantified in samples after deep frying. These were the secoiridoid derivatives, particularly, the isomeric forms of oleuropein and ligstroside aglycone, oleacein, and oleocanthal. Moreover, simple phenols such as hydroxytyrosol and tyrosol were also monitored to correlate them with secoiridoid degradation. Oleocanthalic acid, which has been proposed as a degradation product of oleocanthal, was also determined in each cycle (Supporting Information Tables S3 and S4).^25^ In addition, we studied the significant differences in phenolic compounds between both EVOO cultivars (Arbequina and Picual) in each frying cycle (Supporting Information Tables S5).

Hydroxytyrosol and tyrosol are two simple phenols that are generally found in low concentrations in EVOOs, as they are mainly stated in the pomace.^26,27^ Therefore, an increased concentration of these two phenols in EVOO subjected to deep frying indicates a partial degradation of secoiridoids that are conjugated forms of them. It is worth mentioning that this effect has also been described in EVOOs stored for a one-year period.^9^ In this study, we observed that hydroxytyrosol and tyrosol increased significantly in both EVOOs (p-value: 0.001–0.05) (Figure 4) when the foods were fried during the ten consecutive frying cycles.

**Figure 4:**
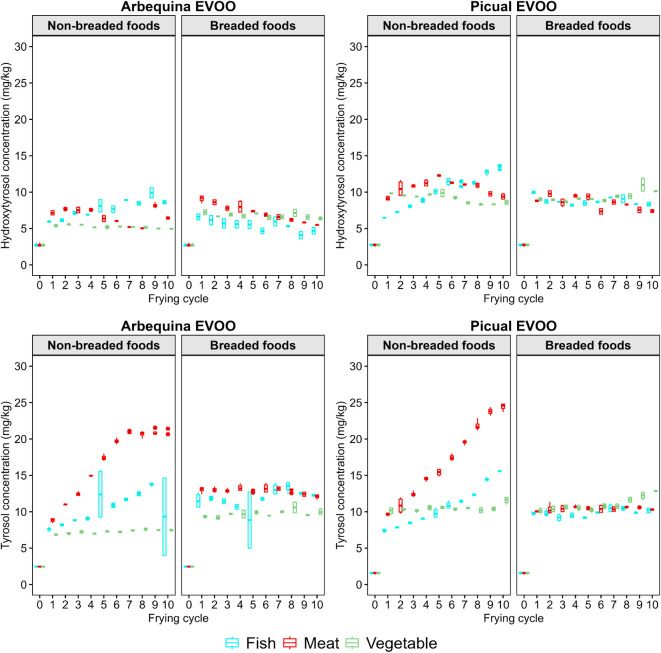
Progressive degradation of hydroxytyrosol and tyrosol in Arbequina and Picual EVOOs during ten frying cycles of nonbreaded foods (red, chicken wings; blue, anchovies; green, potatoes) and breaded foods (red, chicken nuggets; blue, fish fingers; green, eggplants). Concentrations were normalized before statistical analysis.

Concentration levels of hydroxytyrosol were similar by testing breaded and nonbreadcrumbed foods in Picual and Arbequina EVOOs. This phenol increased in the first cycle, and then, the concentration was quite stable around 10 mg/kg. The only exception was the frying of nonbreaded fish in Picual EVOO with a gradual increase of hydroxytyrosol up to 13.1 mg/kg. In the case of tyrosol, clear differences were found, depending on the type of food. Thus, the behavior of tyrosol in breaded foods was similar to that described for hydroxytyrosol. On the other hand, the increase of tyrosol in nonbreaded foods was gradual, up to 24.4 and 21.3 mg/kg for chicken wings fried in Picual and Arbequina EVOOs, respectively, and 15.6 and 9.3 mg/kg for anchovies prepared in the same oils. This difference in nonbreaded foods between hydroxytyrosol and tyrosol can be explained by a higher antioxidant capability and stability of hydroxytyrosol-conjugated secoiridoids as compared to tyrosol derivatives.^28,29^ Potatoes reported similar results to those observed with breaded foods (around 10 mg/kg after the 10th frying cycle).

A similar effect occurred with oleocanthalic acid, which was scarcely detected in Arbequina and Picual EVOOs before frying; however, its concentration was altered as a result of the frying cycle (Figure 5). Thus, the concentration of oleocanthalic acid significantly increased in Arbequina and Picual EVOOs after frying breaded foods, reaching up to 11.9 and 6.9 mg/kg, respectively, in fish fingers, and up to 13.4 and 9.6 mg/kg, respectively, in chicken nuggets. The same pattern observed in breaded foods was found in potatoes (7.6 and 10.6 mg/kg in Picual and Arbequina, respectively). This increase has also been described in EVOO stored for several months.^9^ Nevertheless, a different situation was reported in EVOOs used for frying nonbreaded foods. Thus, we observed a substantial increase in oleocanthalic acid and then a subsequent decay up to residual levels below 2 mg/kg. Moreover, a particular difference was observed between Arbequina and Picual EVOOs, since in the former, the maximum levels of oleocanthalic acid were reported in the second or third frying cycle, whereas in Picual the maximum levels were found in the fifth or sixth cycle. With these results, it seems that a mechanism of oleocanthalic acid decomposition is activated in nonbreaded foods as compared to breaded foods.

**Figure 5:**
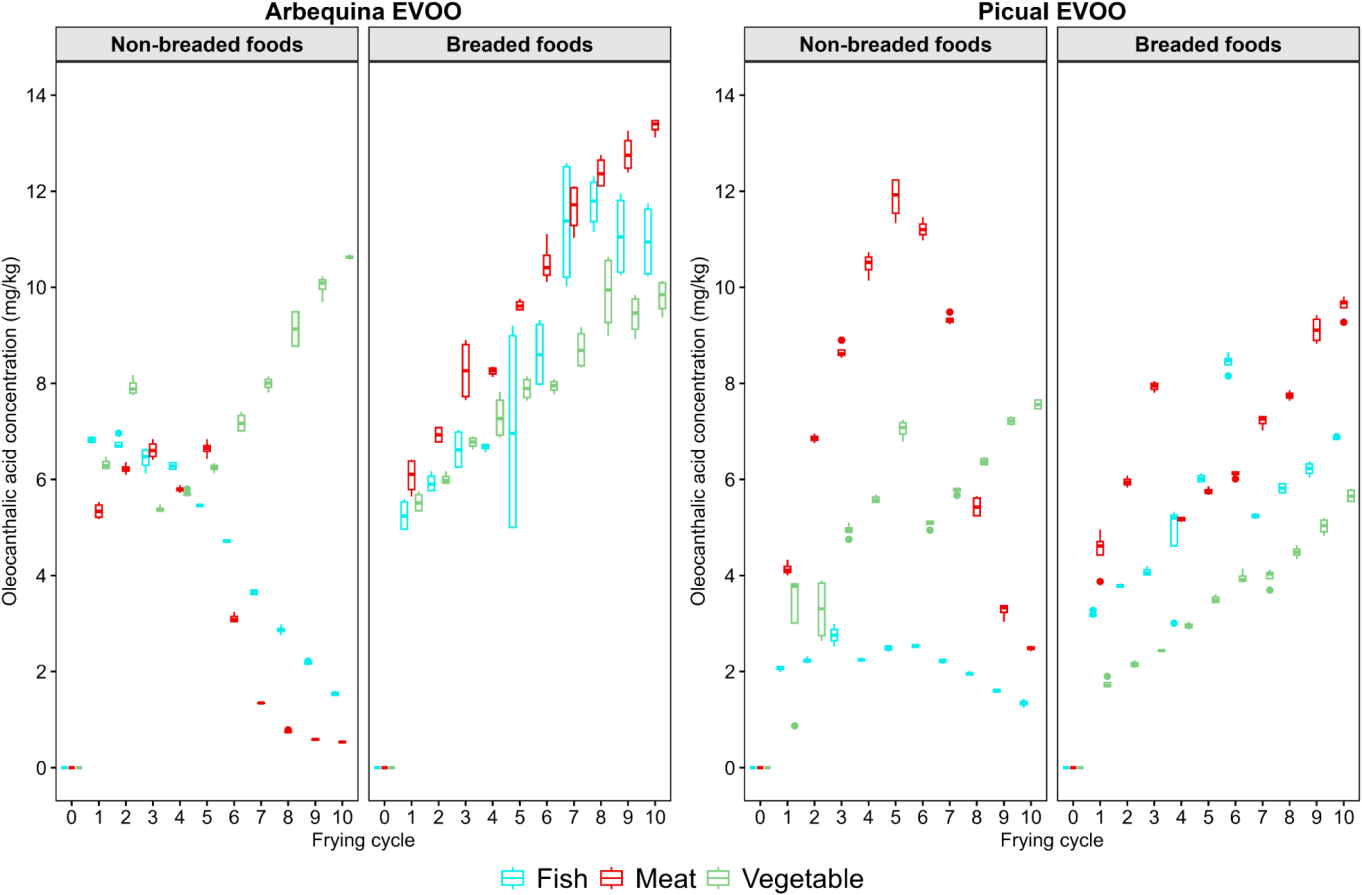
Changes in the concentration of oleocanthalic acid in Arbequina and Picual EVOOs during ten frying cycles of nonbreaded foods (red, chicken wings; blue, anchovies; green, potatoes) and breaded foods (red, chicken nuggets; blue, fish fingers; green, eggplants). Concentrations were normalized before statistical analysis.

Differences were easily visualized when monitoring individual secoiridoid derivatives in processed foods. An important result is that the degradation of the major secoiridoid derivatives (oleuropein and ligstroside aglycones, oleacein, and oleocanthal) in EVOOs is attenuated in potatoes and breaded eggplants. Then, significant differences were also found when comparing breaded and nonbreaded foods during frying. The concentrations of oleuropein and ligstroside aglycones decreased significantly by at least 50% after the first frying cycle in all foods processed in the two EVOOs. Several studies have concluded that the closed forms of oleuropein and ligstroside aglycones possess the highest antioxidant capacity among different phenols found in EVOO.^30–32^ Therefore, these compounds seem to act as the first barrier for olive oil protection, thanks to their antioxidant capability. The degradation rate of these two phenols was similar in relative terms in Arbequina and Picual EVOOs, although in absolute terms, the decrease of these two phenols is quantitatively more relevant in Picual EVOO owing to the higher presence of these phenols in this cultivar. In addition, after the first cycle, concentrations of the two aglycone forms were always higher in breaded foods than in nonbreaded foods, using as reference the same number of frying cycles. Clearly, the decay in the concentration of the two aglycones is more pronounced in nonbreaded foods, which is attributed to the release of the water content of foods without breading (Figure 6).

**Figure 6:**
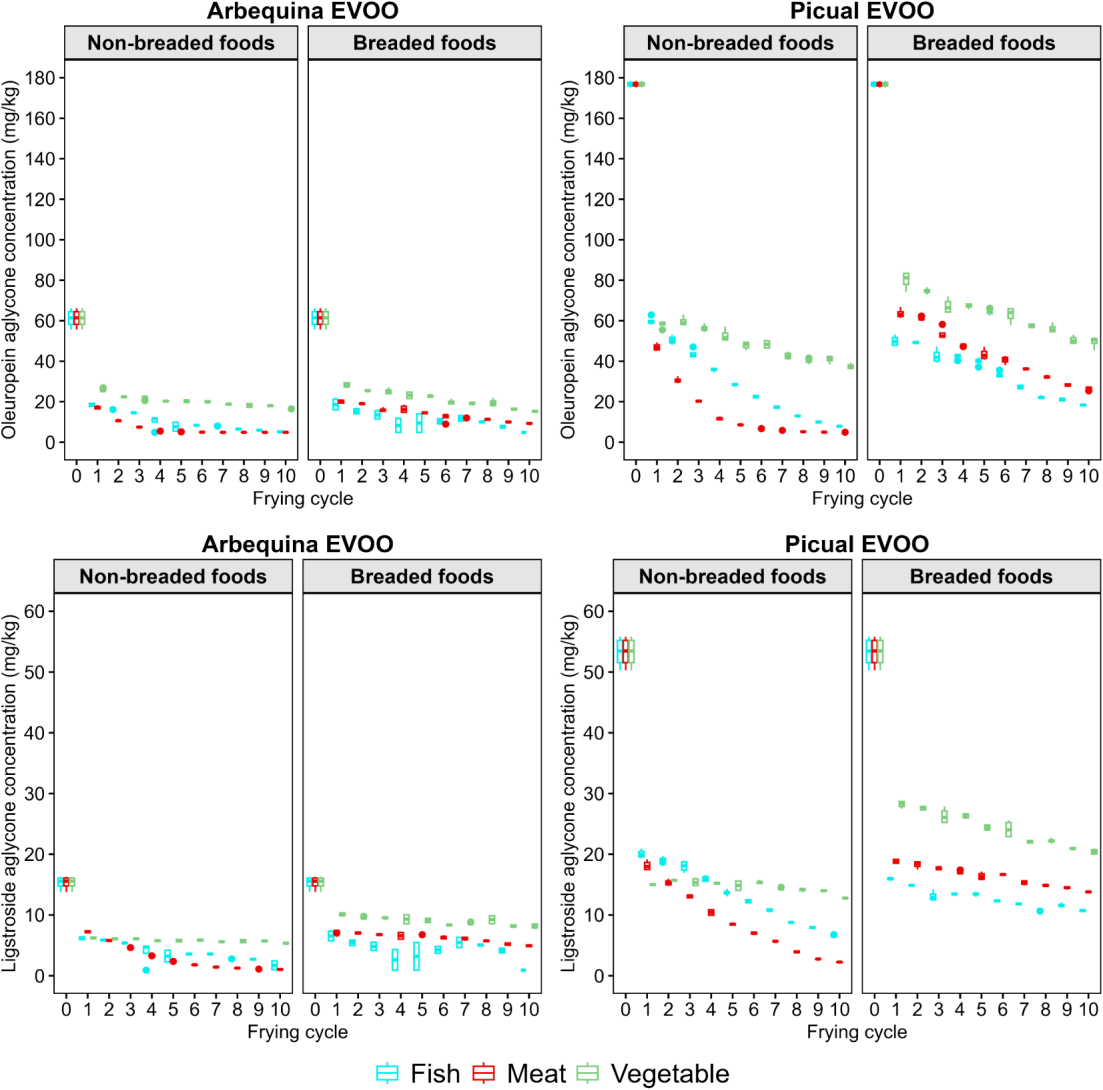
Progressive degradation of the oleuropein and ligstroside aglycone in Arbequina and Picual EVOOs during ten frying cycles of nonbreaded foods (red, chicken wings; blue, anchovies; green, potatoes) and breaded foods (red, chicken nuggets; blue, fish fingers; green, eggplants). Concentrations were normalized before statistical analysis.

These results were complementary to those detected for oleacein and oleocanthal. Thus, we observed a quantitative decrease in the levels of these two phenols in EVOOs subjected to the frying of nonbreaded anchovies and chicken wings. These two phenols were at residual levels after the 10th cycle in these two foods. However, we found some differences between oleacein and oleocanthal concerning the degradation rate in these foods. Particularly, oleacein was at low levels after the fifth and eighth cycles in Arbequina and Picual EVOOs, respectively, whereas oleocanthal was detected at residual levels after the 10th cycle. The results reported for potatoes were like those found for breaded foods and followed the same pattern than breaded eggplant. For these foods, oleacein and oleocanthal were still found at quantitative levels in oils after the 10th frying cycle. Comparing the two monocultivar EVOOs, oleacein experienced a stronger decrease in Arbequina EVOO (especially after the first cycle) used for frying breaded and nonbreaded foods as compared to Picual EVOO. Particularly, this decrease was about 76.8 ± 6.5% and 34.1 ± 10.0% for Arbequina and Picual EVOOs, respectively. Due to the reduced content of oleuropein aglycone in Arbequina EVOO, oleacein acts as the main antioxidant against exposure to high temperatures during the frying process (Figure 7).

**Figure 7:**
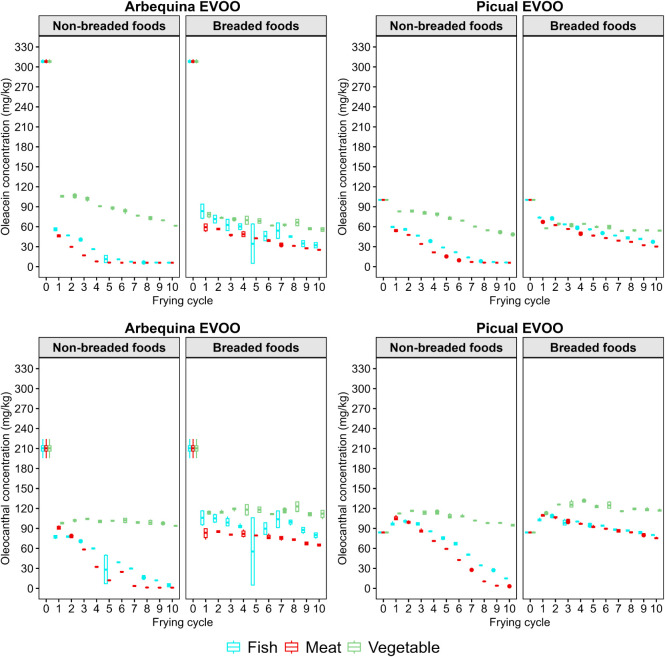
Progressive degradation of the oleacein and oleocanthal in Arbequina and Picual EVOOs during ten frying cycles of nonbreaded foods (red, chicken wings; blue, anchovies; green, potatoes) and breaded foods (red, chicken nuggets; blue, fish fingers; green, eggplants). Concentrations were normalized before statistical analysis.

The result of the oleocanthal degradation after the first cycle was also observed in Arbequina EVOO. However, the levels of this phenol experienced a lower degradation rate compared to oleacein when visualizing the results in Arbequina and Picual EVOOs. In fact, the concentration of this phenol, when frying potatoes and breaded eggplant, was not modified in Arbequina EVOO after the first frying cycle. On the other hand, the concentration of oleocanthal was increased in Picual EVOO after the first frying cycle, and then, it experienced the decrease previously explained. This result can be explained by the high content of oleokoronal in Picual EVOO, which has been previously reported to be converted into oleocanthal when the oil is heated.^33,34^ The same effect could explain the lower decay of oleacein after the first cycle in Picual EVOO as compared to Arbequina EVOO, due to the higher content of oleomissional in the former.

Furthermore, this research also focused on the monoaldehyde open isomers of oleuropein and ligstroside aglycone: oleomissional and oleokoronal, respectively. These isomers are thermolabile compounds. Hence, their concentration experiences a strong decline when olive oil is heated.^22^ The initial oleomissional concentration was 45.6 and 328 mg/kg in Arbequina and Picual EVOOs, respectively, and the oleokoronal concentration was 14.7 and 100 mg/kg in Arbequina and Picual EVOOs, respectively. After the first frying cycle, the oleomissional and oleokoronal content in both EVOO cultivars showed values below 10 mg/kg in EVOOs subjected to frying all of the different foods selected in this research (Supporting Information Figure S2).

### 3.4 Analysis of the Oxidative Stability of EVOOs and Processed Oils

The results reported for the total phenolic content and individual phenols were complemented with the analysis of the oxidative stability of crude and processed oils through the application of the AOCS Cd-12c-16 official method using the Oxitest Oxidation Test Reactor.^20^ The analysis of the unprocessed oils provided clear differences between EVOOs and ordinary olive oil (Table 2). Thus, the oxidative stability of ordinary olive oil was 1.5 h versus 8.0 and 11.6 h for Arbequina and Picual EVOOs, which can be clearly explained by the low phenolic content in the former. Concerning the two EVOOs, the higher response in Picual is explained by its phenolic profile, marked by a predominance in oleuropein and ligstroside aglycones, which have been identified as the main contributors to the oxidative stability of olive oils by Kalaboki et al. (2021).^32^

**Table 2:** Oxidative Stability of Unprocessed and Processed Olive Oils according to the Type of Food[Table-fn tbl2fn1]

**Time (hours)**		
**Unprocessed olive oil**		
Arbequina EVOO	Picual EVOO	Ordinary olive oil
8.0^a^ ± 0.38	11.6^bc^ ± 1.34	1.5^bc^ ± 0.04

	**Processed olive oil**		
	Arbequina EVOO	Picual EVOO	Ordinary olive oil
Non breading			
Potatoes	5.4^bc^ ± 0.04	15.0^ab^ ± 0.01	1.2^d^ ± 0.03
Chicken wings	1.8^e^ ± 0.22	6.8^de^ ± 0.29	0.50^e^ ± 0.05
Anchovies	3.3^de^ ± 0.21	9.8^cd^ ± 0.59	2.1^ab^ ± 0.05
**Breading**			
Eggplants	6.3^ab^ ± 0.13	16.3^a^ ± 0.89	3.3^a^ ± 0.26
Chicken nuggets	3.7^cd^ ± 0.46	6.1^ef^ ± 0.09	1.3^cd^ ± 0.01
Fish fingers	4.8^bcd^ ± 0.52	5.5^f^ ± 0.46	0.53^e^ ± 0.03

i Significant letters refer to the comparison of each oil before and after the 10th frying cycle and were calculated using One-Way ANOVA and Tukey’s HSD.

The oxidative stability significantly decreased when oils were subjected to the ten frying cycles, except for Picual after frying vegetable foods, eggplant, and potato (Table 2). In both cases, the oxidative stability increased after frying 10 cycles as compared with that of crude Picual EVOO. As previously mentioned, the oxidative stability of processed Picual oil is improved because of an enrichment effect of other antioxidant compounds. This effect was not found in Arbequina oil or in ordinary olive oil. Therefore, the phenolic absolute and relative contents of olive oil clearly condition its oxidative stability during frying. Complementarily, the type of food and the use of breading also affect the phenolic degradation, with special emphasis on certain secoiridoid derivatives.

## Supplementary Material



## Notes

LC–MS/MS parameters for the determination of phenols (Table S1); calibration models obtained for the quantitative analysis of phenolic compounds in olive oil (Table S2); mean concentrations (mg/kg) and standard deviations (SD) of the phenolic compounds quantified in Arbequina oil (Table S3); mean concentrations (mg/kg) and standard deviations (SD) of the phenolic compounds quantified in Picual oil (Table S4); significant differences obtained between the phenolic compounds of each food EVOO according to the cultivar (Arbequina and Picual) and frying cycle by pairwise comparisons (Table S5); chemical structures of the main secoiridoid derivatives found in olive oil (Figure S1); progressive degradation of oleomissional and oleokoronal in Arbequina and Picual EVOOs (Figure S2) (PDF)

## Abbreviations

3,4-DHPEA-EDA: Oleacein
EFSA: European Food Safety Authority
EVOO: Extra Virgin Olive Oil
IS: Internal Standard
OO: Olive Oil
p-HPEA-EDA: Oleocanthal
VOO: Virgin Olive Oil
