# Supplementary material for: Deep Frying in Extra-Virgin Olive Oil: Evaluating the Influence of the Type of Food and Breading on the Degradation of Phenolic Compounds
Source: J Agric Food Chem. 2026 May 27;74(22):17088–98. doi: 10.1021/acs.jafc.6c00555 (PMC13266970; doi:10.1021/acs.jafc.6c00555)
Supplement: Supplementary file 1 [file jf6c00555_si_001.pdf]

Deep frying in extra-virgin olive oil: evaluating the influence of the type of food and breading on the degradation of phenolic compounds

Ana Castillo-Luna[a,b], Feliciano Priego-Capote[a,b,c,d*]

[a]Department of Analytical Chemistry, Campus of Rabanales, University of Córdoba, Córdoba, 14014, Spain.

[b]Chemical Institute for Energy and Environment (IQUEMA), Campus of Rabanales, University of Córdoba, Córdoba, 14014, Spain.

[c]Maimónides Institute of Biomedical Research (IMIBIC), Reina Sofía University Hospital, University of Córdoba, Córdoba, 14014, Spain.

[d]Consortium for Biomedical Research in Frailty & Healthy Ageing, CIBERFES, Carlos III Institute of Health, Madrid, 28029, Spain.

*Corresponding author: F. Priego-Capote (q72prcaf@uco.es).

Phone and fax: +34957218615.

Supplementary Table S1. LC–MS/MS parameters for determination of phenols.

Compound	RT* (min)	Precursor ion (m/z)	Product ion (m/z)	Fragmentor	Collision energy (eV)
Oleocanthalic acid	1.5	319.2	111.0	80	15
Hydroxytyrosol	2.0	153.1	123.1	40	15
Tyrosol	2.2	137.1	106.1	80	15
Syringaldehyde (IS)	2.7	181.1	151.1	40	15
Oleacein	3.4	319.2	69.3	80	20
Oleomissional	3.6	377.2	275.1	20	20
Oleocanthal	5.3	303.1	59.3	80	15
Oleokoronal	5.5	361.1	291.1	60	15
Oleuropein aglycone	6.0	377.2	275.1	20	20
Ligstroside aglycone	6.6	361.1	291.1	60	15

**Retention time.*

Supplementary Table S2. Calibration models obtained for quantitative analysis of phenolic compounds in olive oil.

Compound	Calibration model	R^2*	Calibration range
Hydroxytyrosol	$y = 6.3626x \pm 0.0393 - 0.3796 \pm 0.0913$	0.9997	1 – 20 mg/kg
Tyrosol	$y = 0.2806x \pm 0.0029 - 0.0263 \pm 0.0067$	0.9992	1 – 20 mg/kg
Oleacein	$y = 1.7773x \pm 0.0332 - 0.2653 \pm 0.0773$	0.9972	1 – 20 mg/kg
Oleocanthal	$y = 2.7366x \pm 0.0399 - 0.1488 \pm 0.0929$	0.9983	1 – 20 mg/kg
Oleuropein aglycone	$y = 1.2930x \pm 0.0200 - 0.1574 \pm 0.0453$	0.9981	1 – 20 mg/kg
Ligstroside aglycone	$y = 15.8647x \pm 0.1125 - 0.3640 \pm 0.2615$	0.9996	1 – 20 mg/kg

**Regression coefficient.*

Supplementary Table S3. Mean concentrations (mg/kg) and standard deviations (SD) of the phenolic compounds quantified in Arbequina olive oil samples obtained after frying different foods during ten cycles.

Frying cycle	Hydroxytyrosol		Tyrosol		Oleocanthalic acid		Oleuropein aglycone		Oleomissional		Ligstroside aglycone		Oleokoronal		Oleacein		Oleocanthal		Total phenols
**Anchovies**																			
	Mean	SD	Mean	SD	Mean	SD	Mean	SD	Mean	SD	Mean	SD	Mean	SD	Mean	SD	Mean	SD	
0	2.7	0.23	2.5	0.08	0.00	0.00	61.2	4.74	46.4	3.4	15.3	1.1	16.2	1.4	308	2.6	210	11.9	662
1st	6.0	0.15	7.6	0.30	6.8	0.07	18.5	0.84	6.5	0.14	6.2	0.29	1.8	0.03	56.2	2.5	77.3	2.7	187
2nd	6.1	0.22	8.2	0.19	6.8	0.14	16.7	0.43	5.9	0.16	5.9	0.13	1.3	0.03	46.9	0.46	77.6	1.5	175
3rd	7.1	0.26	8.8	0.12	6.4	0.24	14.6	0.38	5.8	0.09	5.4	0.06	1.3	0.02	39.5	0.81	72.0	0.86	161
4th	6.9	0.05	8.9	0.11	6.3	0.08	10.1	3.48	5.3	0.28	3.8	1.9	1.1	0.14	26.1	0.66	59.8	1.2	128
5th	8.1	1.00	12.4	3.67	5.5	0.03	7.7	2.52	5.1	0.24	3.2	1.1	1.1	0.09	11.6	6.3	28.6	24.9	83.3
6th	7.7	0.52	10.7	0.18	4.7	0.03	8.4	0.22	5.0	0.05	3.6	0.10	1.1	0.02	11.1	0.09	39.3	0.63	91.6
7th	8.9	0.08	11.7	0.23	3.6	0.06	7.8	0.15	0.00	0.00	3.6	0.09	1.0	0.01	7.5	0.10	29.7	1.0	73.8
8th	8.5	0.32	12.3	0.18	2.9	0.09	6.5	0.15	0.00	0.00	3.0	0.14	1.0	0.01	6.4	0.03	18.0	1.4	58.6
9th	9.9	0.85	13.8	0.24	2.2	0.03	5.9	0.16	0.00	0.00	2.7	0.04	1.0	0.00	6.2	0.01	12.1	0.17	53.8
10th	8.6	0.26	9.3	6.14	1.5	0.05	5.1	0.25	0.00	0.00	1.7	0.85	1.0	0.02	6.0	0.04	4.8	2.8	38.0
**Chicken wings**																			
**Frying cycle**	**Hydroxytyrosol**		**Tyrosol**		**Oleocanthalic acid**		**Oleuropein aglycone**		**Oleomissional**		**Ligstroside aglycone**		**Oleokoronal**		**Oleacein**		**Oleocanthal**		**Total phenols**
	Mean	SD	Mean	SD	Mean	SD	Mean	SD	Mean	SD	Mean	SD	Mean	SD	Mean	SD	Mean	SD	
0	2.7	0.23	2.5	0.08	0.00	0.00	61.2	4.74	46.4	3.43	15.3	1.1	16.2	1.4	308	2.6	210	11.9	662

1st	7.2	0.35	8.8	0.30	5.3	0.17	17.2	0.92	6.1	0.12	7.2	0.18	1.2	0.03	46.4	2.2	91.2	2.9	191
2nd	7.8	0.10	11.0	0.08	6.2	0.11	10.7	0.67	5.5	0.06	5.8	0.11	1.1	0.01	29.9	0.49	77.0	1.4	155
3rd	7.6	0.47	12.5	0.28	6.6	0.19	7.5	0.15	5.1	0.04	4.5	0.10	1.0	0.01	17.0	0.49	58.3	0.82	120
4th	7.7	0.08	15.0	0.03	5.8	0.07	5.6	0.10	0.00	0.00	3.4	0.05	1.0	0.01	7.9	0.13	32.4	0.51	78.8
5th	6.4	0.51	17.4	0.43	6.6	0.17	5.1	0.01	0.00	0.00	2.3	0.03	1.0	0.01	6.1	0.01	12.2	0.30	57.1
6th	6.0	0.05	19.7	0.38	3.1	0.10	5.0	0.02	0.00	0.00	1.8	0.02	1.0	0.01	5.7	0.19	25.0	0.63	67.3
7th	5.2	0.07	20.9	0.16	1.3	0.01	4.9	0.02	0.00	0.00	1.4	0.07	1.0	0.00	4.5	0.12	3.6	0.14	42.8
8th	5.0	0.03	20.7	0.45	0.75	0.02	4.9	0.01	0.00	0.00	1.3	0.02	0.94	0.00	4.1	0.06	1.3	0.08	39.0
9th	8.2	0.36	20.9	0.43	0.58	0.02	4.9	0.01	0.00	0.00	1.1	0.01	0.94	0.00	2.9	0.06	1.0	0.04	40.5
10th	6.4	0.22	21.3	0.48	0.53	0.02	4.9	0.01	0.0	0.00	1.0	0.01	0.94	0.00	2.0	0.03	1.0	0.05	38.1
**Potatoes**																			
**Frying cycle**	**Hydroxytyrosol**		**Tyrosol**		**Oleocanthalic acid**		**Oleuropein aglycone**		**Oleomissional**		**Ligstroside aglycone**		**Oleokoronal**		**Oleacein**		**Oleocanthal**		**Total phenols**
	**Mean**	**SD**	**Mean**	**SD**	**Mean**	**SD**	**Mean**	**SD**	**Mean**	**SD**	**Mean**	**SD**	**Mean**	**SD**	**Mean**	**SD**	**Mean**	**SD**	
0	2.7	0.23	2.5	0.08	0.00	0.00	61.2	4.74	46.4	3.4	15.3	1.1	16.2	1.4	308	2.6	210	11.9	662
1st	5.4	0.20	6.9	0.16	6.3	0.12	25.7	0.77	8.6	0.15	6.2	0.22	2.8	0.08	106	1.8	98.0	1.8	266
2nd	5.6	0.13	7.0	0.19	7.9	0.19	22.3	0.57	7.7	0.41	6.1	0.12	1.9	0.02	104	2.0	102	2.5	265
3rd	5.5	0.04	7.2	0.19	5.4	0.07	21.9	0.94	7.1	0.04	6.1	0.09	1.6	0.02	102	3.5	104	1.5	261
4th	5.2	0.04	7.0	0.14	5.7	0.07	20.2	0.25	6.7	0.10	5.8	0.14	1.4	0.01	90.7	1.1	100	2.4	243
5th	5.1	0.05	7.3	0.12	6.2	0.09	20.2	0.76	6.8	0.17	5.8	0.22	1.3	0.04	88.0	2.7	102	1.1	243
6th	5.3	0.12	7.2	0.18	7.2	0.21	19.9	0.63	6.8	0.27	5.9	0.20	1.3	0.03	83.8	3.8	102	4.0	239
7th	5.2	0.10	7.4	0.14	8.0	0.14	18.8	0.59	6.6	0.08	5.6	0.09	1.2	0.02	76.5	0.80	99.0	1.8	228

8th	5.2	0.16	7.6	0.27	9.1	0.42	18.2	1.06	6.5	0.14	5.7	0.28	1.3	0.01	73.0	3.0	99.2	3.9	226
9th	5.0	0.02	7.5	0.05	10.0	0.24	17.9	0.50	6.4	0.19	5.7	0.10	1.3	0.02	69.5	0.83	98.4	0.52	222
10th	5.0	0.08	7.5	0.16	10.6	0.04	16.2	0.18	6.4	0.11	5.4	0.06	1.3	0.01	61.5	0.95	94.0	1.3	208

Fish fingers

Frying cycle	Hydroxytyrosol		Tyrosol		Oleocanthalic acid		Oleuropein aglycone		Oleomissional		Ligstroside aglycone		Oleokoronal		Oleacein		Oleocanthal		Total phenols
	Mean	SD	Mean	SD	Mean	SD	Mean	SD	Mean	SD	Mean	SD	Mean	SD	Mean	SD	Mean	SD	
0	2.7	0.23	2.5	0.08	0.00	0.00	61.2	4.74	46.4	3.4	15.3	1.1	16.2	1.4	308	2.6	210	11.9	662
1st	6.6	0.49	11.5	1.08	5.3	0.34	18.7	3.30	7.7	0.37	6.5	0.97	2.0	0.14	83.2	12.7	106	12.5	248
2nd	6.2	0.77	12.0	0.18	5.9	0.21	15.3	1.68	7.1	0.36	5.4	0.55	1.6	0.07	71.4	6.8	105	6.9	230
3rd	5.7	0.75	11.4	0.38	6.6	0.43	13.6	2.51	6.5	0.33	4.8	0.79	1.4	0.06	62.7	9.8	99.9	7.2	213
4th	5.1	0.04	10.4	0.21	6.7	0.09	8.4	4.11	5.6	0.83	2.6	2.0	1.1	0.26	55.9	1.0	91.0	0.61	187
5th	6.1	0.02	12.7	0.03	9.0	0.12	9.4	5.26	5.6	1.07	3.2	2.6	1.2	0.29	64.6	0.56	106	0.52	218
6th	4.7	0.47	11.8	0.27	8.6	0.74	10.3	1.64	6.0	0.24	4.3	0.65	1.3	0.06	46.1	8.3	89.8	10.5	183
7th	6.0	0.73	13.1	0.80	11.3	1.38	11.8	1.66	6.0	0.27	5.5	0.99	1.3	0.04	54.1	13.5	104	14.5	213
8th	5.3	0.15	13.4	0.77	11.8	0.56	10.0	0.34	5.7	0.06	5.1	0.20	1.3	0.03	45.2	1.73	99.4	4.2	197
9th	4.1	0.59	12.5	0.20	11.1	0.90	7.6	0.73	5.5	0.11	4.2	0.45	1.3	0.02	34.6	5.3	87.5	5.2	168
10th	3.9	0.13	13.3	0.69	11.9	0.41	4.9	0.03	4.9	0.01	0.92	0.00	0.92	0.00	35.0	0.65	82.7	0.35	158

Chicken nuggets																				
Frying cycle	Hydroxytyrosol		Tyrosol		Oleocanthalic acid		Oleuropein aglycone		Oleomissional		Ligstroside aglycone		Oleokoronal		Oleacein		Oleocanthal		Total phenols
	Mean	SD	Mean	SD	Mean	SD	Mean	SD	Mean	SD	Mean	SD	Mean	SD	Mean	SD	Mean	SD	
0	2.7	0.23	2.5	0.08	0.00	0.00	61.2	4.74	46.4	3.4	15.3	1.1	16.2	1.4	308	2.6	210	11.9	662
1st	9.1	0.49	13.0	0.44	6.1	0.38	20.1	0.98	6.3	0.22	7.3	0.18	1.3	0.16	58.9	6.4	82.5	8.4	205
2nd	8.6	0.43	13.0	0.37	6.9	0.18	19.0	0.36	5.8	0.14	7.0	0.22	1.1	0.01	56.5	1.5	85.3	1.6	203
3rd	7.8	0.38	12.9	0.32	8.3	0.66	16.0	0.99	5.7	0.12	6.8	0.15	1.0	0.02	47.2	1.7	80.7	1.3	186
4th	8.0	0.89	13.3	0.48	8.3	0.10	16.4	1.95	5.8	0.19	6.6	0.66	1.1	0.06	49.0	4.5	82.2	5.6	191
5th	7.4	0.13	13.0	0.25	9.6	0.11	14.6	0.49	5.5	0.06	6.7	0.07	1.0	0.01	42.4	0.84	79.3	0.83	180
6th	6.9	0.29	13.2	0.65	10.5	0.43	12.3	2.23	5.4	0.08	6.3	0.33	1.0	0.02	39.3	1.9	77.6	3.7	173
7th	6.6	0.40	13.2	0.30	11.6	0.53	12.5	0.44	5.3	0.08	6.1	0.29	1.0	0.01	34.3	1.5	75.5	3.1	166
8th	6.2	0.25	13.0	0.27	12.4	0.33	11.2	0.36	5.3	0.08	5.7	0.12	1.0	0.01	31.1	0.31	73.2	1.4	159
9th	5.9	0.06	12.4	0.33	12.8	0.41	10.0	0.36	5.3	0.10	5.2	0.20	1.0	0.00	27.7	0.80	67.4	2.6	148
10th	5.5	0.04	12.1	0.31	13.4	0.17	9.3	0.29	5.3	0.08	4.9	0.17	1.0	0.01	25.2	0.69	65.0	2.0	142

Eggplants																				
Frying cycle	Hydroxytyrosol		Tyrosol		Oleocanthalic acid		Oleuropein aglycone		Oleomissional		Ligstroside aglycone		Oleokoronal		Oleacein		Oleocanthal		Total phenols
	Mean	SD	Mean	SD	Mean	SD	Mean	SD	Mean	SD	Mean	SD	Mean	SD	Mean	SD	Mean	SD	
0	2.7	0.23	2.5	0.08	0.00	0.00	61.2	4.74	46.4	3.4	15.3	1.1	16.2	1.4	308	2.6	210	11.9	662
1st	7.3	0.40	9.3	0.22	5.5	0.22	28.3	1.45	7.5	0.25	10.1	0.33	2.6	0.14	77.9	4.5	114	3.0	263
2nd	6.7	0.06	9.2	0.30	6.0	0.12	25.5	0.45	6.2	0.15	9.5	0.22	1.5	0.04	73.6	1.4	115	2.6	253

3rd	6.9	0.27	9.7	0.13	6.8	0.12	25.1	1.50	6.1	0.10	9.5	0.24	1.2	0.03	72.6	1.1	119	2.5	257
4th	6.7	0.33	9.7	0.63	7.3	0.47	23.2	1.88	6.0	0.10	9.3	0.88	1.1	0.01	69.8	7.0	118	9.3	251
5th	7.1	0.06	9.9	0.29	7.9	0.25	22.8	0.89	6.0	0.11	9.1	0.43	1.1	0.02	68.6	4.1	118	4.8	251
6th	6.5	0.34	9.5	0.04	7.9	0.14	19.7	1.30	5.9	0.09	8.4	0.13	1.1	0.01	61.8	0.52	112	1.2	233
7th	6.6	0.33	10.0	0.18	8.7	0.42	19.2	0.89	5.8	0.11	8.6	0.17	1.1	0.02	62.7	2.1	115	2.4	238
8th	7.1	0.57	10.6	0.87	9.9	0.83	19.5	1.92	5.7	0.20	9.2	0.79	1.1	0.03	66.3	5.9	123	8.6	252
9th	6.6	0.39	9.5	0.13	9.4	0.44	16.4	0.58	5.6	0.11	8.2	0.24	1.1	0.01	57.1	2.1	111	3.2	225
10th	6.4	0.02	10.0	0.49	9.8	0.37	15.3	0.51	5.6	0.06	8.2	0.43	1.1	0.01	55.7	3.4	112	6.8	224

Supplementary Table S4. Mean concentrations (mg/kg) and standard deviations (SD) of the phenolic compounds quantified in Picual olive oil samples obtained after frying different foods during ten cycles.

Frying cycle	Hydroxytyrosol		Tyrosol		Oleocanthalic acid		Oleuropein aglycone		Oleomissional		Ligstroside aglycone		Oleokoronal		Oleacein		Oleocanthal		Total phenols
**Anchovies**																			
	Mean	SD	Mean	SD	Mean	SD	Mean	SD	Mean	SD	Mean	SD	Mean	SD	Mean	SD	Mean	SD	
0	2.8	0.20	1.58	0.17	0.00	0.00	181	4.9	327	1.5	53.3	2.6	99.5	1.3	100	0.11	84.1	1.3	849
1st	6.5	0.06	7.4	0.24	2.1	0.05	59.8	2.1	9.8	0.15	20.1	0.70	4.5	0.08	59.6	1.2	96.9	2.7	267
2nd	7.3	0.12	7.9	0.12	2.2	0.06	50.4	2.2	7.7	0.25	19.2	0.42	1.9	0.02	56.0	2.0	101	2.7	254
3rd	8.1	0.23	8.5	0.18	2.8	0.20	43.6	2.3	7.4	0.19	18.0	0.95	1.6	0.05	46.7	1.3	96.9	2.5	234
4th	8.7	0.18	9.1	0.15	2.3	0.03	35.8	0.88	7.0	0.11	16.0	0.53	1.5	0.02	39.2	0.47	85.7	1.3	205
5th	10.1	0.34	9.8	0.67	2.5	0.06	28.4	0.68	6.7	0.27	13.7	0.51	1.5	0.12	28.7	0.74	75.4	2.7	177
6th	11.4	0.55	10.9	0.52	2.5	0.03	22.5	0.96	6.3	0.27	12.3	0.33	1.5	0.06	21.7	0.16	67.2	2.4	156
7th	10.9	0.41	11.4	0.24	2.2	0.04	17.3	0.66	5.9	0.07	10.8	0.27	1.4	0.01	13.9	0.09	50.9	1.4	125
8th	11.3	0.23	12.3	0.17	2.0	0.04	13.0	0.28	5.3	0.09	8.8	0.09	1.3	0.01	8.3	0.09	34.6	0.33	96.9
9th	12.7	0.41	14.4	0.27	1.6	0.04	10.1	0.48	5.2	0.05	7.9	0.16	1.3	0.05	6.9	0.03	26.8	0.35	86.9
10th	13.1	0.31	15.6	0.09	1.3	0.07	7.9	0.19	4.9	0.05	6.6	0.15	1.2	0.01	6.3	0.02	15.0	0.21	71.9
**Chicken wings**																			
Frying cycle	Hydroxytyrosol		Tyrosol		Oleocanthalic acid		Oleuropein aglycone		Oleomissional		Ligstroside aglycone		Oleokoronal		Oleacein		Oleocanthal		Total phenols
	Mean	SD	Mean	SD	Mean	SD	Mean	SD	Mean	SD	Mean	SD	Mean	SD	Mean	SD	Mean	SD	
0	2.8	0.20	1.58	0.17	0.00	0.00	181	4.9	327	1.5	53.3	2.6	99.5	1.3	100	0.11	84.1	1.3	849

1st	9.2	0.32	9.7	0.23	4.5	0.46	47.0	1.9	7.8	0.22	18.2	0.82	1.6	0.11	54.2	2.3	106	3.5	258
2nd	10.0	1.71	10.9	1.13	6.0	0.10	30.6	1.5	7.0	0.40	15.3	0.53	1.6	0.23	47.8	0.41	99.3	2.5	229
3rd	10.9	0.21	12.4	0.37	7.9	0.11	20.2	0.54	6.0	0.11	13.1	0.30	1.4	0.00	34.0	0.58	86.4	2.6	192
4th	11.3	0.50	14.5	0.30	5.2	0.05	11.7	0.74	5.6	0.06	10.4	0.55	1.4	0.08	21.6	0.80	71.2	0.94	153
5th	12.3	0.18	15.4	0.51	5.8	0.08	8.6	0.25	5.3	0.06	8.5	0.13	1.3	0.08	15.7	0.23	59.3	0.57	132
6th	11.3	0.13	17.5	0.46	6.1	0.07	6.8	0.08	5.0	0.01	7.0	0.26	1.2	0.04	9.9	0.19	42.7	0.34	108
7th	11.0	0.16	19.6	0.34	7.2	0.13	5.7	0.10	4.9	0.01	5.7	0.07	1.2	0.01	7.1	0.05	27.3	0.35	89.7
8th	10.9	0.37	21.8	0.73	7.7	0.09	5.2	0.05	4.9	0.04	3.9	0.13	1.1	0.00	6.1	0.01	10.5	0.12	72.1
9th	9.8	0.35	23.8	0.51	9.1	0.29	5.0	0.01	4.6	0.04	2.8	0.04	1.0	0.00	4.2	0.08	4.0	0.27	64.3
10th	9.4	0.45	24.4	0.53	9.6	0.23	4.9	0.01	4.4	0.02	2.2	0.05	1.0	0.01	3.1	0.07	3.2	0.03	62.2
**Potatoes**																			
**Frying cycle**	**Hydroxytyrosol**		**Tyrosol**		**Oleocanthalic acid**		**Oleuropein aglycone**		**Oleomissional**		**Ligstroside aglycone**		**Oleokoronal**		**Oleacein**		**Oleocanthal**		**Total phenols**
	**Mean**	**SD**	**Mean**	**SD**	**Mean**	**SD**	**Mean**	**SD**	**Mean**	**SD**	**Mean**	**SD**	**Mean**	**SD**	**Mean**	**SD**	**Mean**	**SD**	
0	2.8	0.20	1.58	0.17	0.00	0.00	181	4.9	327	1.5	53.3	2.6	99.5	1.3	100	0.11	84.1	1.3	849
1st	9.8	0.07	10.1	0.51	3.1	1.46	58.2	1.8	10.4	0.49	15.0	0.12	2.1	0.12	82.9	0.86	113	1.3	305
2nd	9.5	0.10	10.3	0.19	3.3	0.67	59.6	2.4	10.3	0.28	15.7	0.18	2.1	0.01	83.5	1.9	117	1.2	311
3rd	9.4	0.02	10.1	0.24	4.9	0.14	56.3	1.9	9.8	0.28	15.4	0.70	2.0	0.04	80.8	2.7	114	3.1	303
4th	9.7	0.16	10.4	0.15	5.6	0.09	52.8	3.2	9.9	0.30	15.2	0.23	2.0	0.02	78.7	2.9	113	1.7	297
5th	9.9	0.62	10.6	0.20	7.0	0.20	47.8	1.9	9.5	0.69	14.8	0.92	2.0	0.08	72.9	2.6	109	3.6	284
6th	9.2	0.25	10.4	0.23	5.1	0.09	48.4	2.1	8.8	0.22	15.4	0.31	1.9	0.05	69.4	1.3	109	2.2	278
7th	8.5	0.19	10.5	0.15	5.8	0.07	42.6	1.8	9.0	0.21	14.3	0.17	2.0	0.05	60.4	0.74	102	0.79	255

8th	8.3	0.19	10.2	0.43	6.4	0.08	40.0	1.2	8.2	0.57	14.1	0.25	1.9	0.12	54.8	1.1	98.3	1.2	242
9th	8.3	0.08	10.5	0.12	7.2	0.09	41.1	2.0	8.3	0.19	14.0	0.18	1.9	0.01	52.9	0.86	98.3	0.71	243
10th	8.6	0.34	11.6	0.49	7.6	0.11	37.4	1.4	8.9	0.14	12.8	0.04	2.1	0.08	47.8	0.46	95.0	1.7	232
**Fish fingers**																			
**Frying cycle**	**Hydroxytyrosol**		**Tyrosol**		**Oleocanthalic acid**		**Oleuropein aglycone**		**Oleomissional**		**Ligstroside aglycone**		**Oleokoronal**		**Oleacein**		**Oleocanthal**		**Total phenols**
	**Mean**	**SD**	**Mean**	**SD**	**Mean**	**SD**	**Mean**	**SD**	**Mean**	**SD**	**Mean**	**SD**	**Mean**	**SD**	**Mean**	**SD**	**Mean**	**SD**	
0	2.8	0.20	1.58	0.17	0.00	0.00	181	4.9	327	1.5	53.3	2.6	99.5	1.3	100	0.11	84.1	1.3	849
1st	10.0	0.23	9.8	0.20	3.2	0.07	50.0	2.7	10.7	0.46	16.0	0.31	3.9	0.13	74.1	1.1	103	2.5	281
2nd	8.7	0.32	9.8	0.49	3.8	0.04	49.3	0.60	9.3	0.42	14.9	0.12	2.4	0.08	71.2	1.1	107	1.6	276
3rd	8.7	0.08	9.2	0.55	4.1	0.09	42.6	3.6	8.5	0.43	13.1	0.84	1.9	0.09	63.7	1.2	99.1	4.8	251
4th	8.2	0.19	9.5	0.39	4.7	1.12	42.3	1.4	8.1	0.04	13.4	0.14	1.8	0.05	59.8	1.2	100	0.62	248
5th	8.5	0.39	9.2	0.13	6.0	0.09	39.9	2.0	7.6	0.41	13.4	0.36	1.6	0.06	55.8	1.4	96.5	1.7	239
6th	8.6	0.15	9.9	0.14	8.4	0.21	33.2	1.7	7.1	0.16	12.4	0.20	1.5	0.05	51.3	0.58	94.1	1.2	227
7th	9.3	0.03	10.5	0.58	5.2	0.05	27.3	0.98	7.0	0.25	11.8	0.17	1.5	0.03	46.8	0.61	88.1	1.5	208
8th	9.1	0.65	10.6	0.14	5.8	0.11	22.2	0.75	6.7	0.22	10.9	0.16	1.4	0.01	43.3	1.9	86.7	1.4	197
9th	8.4	0.08	9.9	0.15	6.2	0.15	21.1	0.75	5.8	0.10	11.6	0.36	1.2	0.03	41.7	1.2	83.9	2.2	190
10th	8.4	0.33	10.2	0.39	6.9	0.02	18.3	0.39	5.6	0.09	10.7	0.15	1.2	0.07	37.8	0.32	80.0	0.70	179

Chicken nuggets																			
**Frying cycle**	**Hydroxytyrosol**		**Tyrosol**		**Oleocanthalic acid**		**Oleuropein aglycone**		**Oleomissional**		**Ligstroside aglycone**		**Oleokoronal**		**Oleacein**		**Oleocanthal**		**Total phenols**
	**Mean**	**SD**	**Mean**	**SD**	**Mean**	**SD**	**Mean**	**SD**	**Mean**	**SD**	**Mean**	**SD**	**Mean**	**SD**	**Mean**	**SD**	**Mean**	**SD**	
0	2.8	0.20	1.58	0.17	0.00	0.00	181	4.9	327	1.5	53.3	2.6	99.5	1.3	100	0.11	84.1	1.3	849
1st	8.8	0.15	10.1	0.14	4.5	0.46	63.5	2.4	8.6	0.11	18.8	0.39	1.7	0.02	68.2	0.67	110	1.8	294
2nd	9.8	0.48	10.3	0.72	6.0	0.10	61.0	0.83	8.4	0.55	18.3	0.60	1.6	0.14	62.3	1.2	107	1.8	285
3rd	8.6	0.57	10.4	0.51	7.9	0.11	53.6	3.1	8.0	0.24	17.7	0.31	1.6	0.09	56.5	0.70	102	1.8	266
4th	9.5	0.25	10.7	0.23	5.2	0.05	47.9	0.45	7.6	0.20	17.0	0.29	1.6	0.03	51.0	1.2	97.2	1.4	248
5th	9.4	0.48	10.5	0.30	5.8	0.08	43.4	2.9	7.2	0.24	16.4	0.65	1.6	0.11	46.9	1.1	92.7	2.0	234
6th	7.3	0.46	10.4	0.58	6.1	0.07	40.6	2.0	6.7	0.37	16.6	0.17	1.4	0.08	43.1	0.29	89.3	1.3	222
7th	8.6	0.34	10.4	0.39	7.2	0.13	36.3	0.42	6.7	0.43	15.3	0.39	1.4	0.07	39.0	1.1	86.4	2.4	211
8th	8.3	0.11	10.6	0.19	7.7	0.09	32.4	0.73	6.7	0.04	14.9	0.25	1.5	0.04	37.2	0.80	84.0	1.4	203
9th	7.6	0.46	10.5	0.04	9.1	0.29	28.2	0.69	6.2	0.16	14.5	0.23	1.4	0.02	32.3	0.38	81.4	1.1	191
10th	7.4	0.25	10.3	0.19	9.6	0.23	26.6	0.91	5.8	0.16	13.8	0.17	1.3	0.04	30.3	0.54	75.7	1.4	181

Eggplants																			
**Frying cycle**	**Hydroxytyrosol**		**Tyrosol**		**Oleocanthalic acid**		**Oleuropein aglycone**		**Oleomissional**		**Ligstroside aglycone**		**Oleokoronal**		**Oleacein**		**Oleocanthal**		**Total phenols**
	**Mean**	**SD**	**Mean**	**SD**	**Mean**	**SD**	**Mean**	**SD**	**Mean**	**SD**	**Mean**	**SD**	**Mean**	**SD**	**Mean**	**SD**	**Mean**	**SD**	
0	2.8	0.20	1.58	0.17	0.00	0.00	181	4.9	327	1.5	53.3	2.6	99.5	1.3	100	0.11	84.1	1.3	849
1st	8.9	0.06	10.2	0.40	1.7	0.10	80.1	4.5	13.0	0.26	28.2	0.63	8.7	0.25	57.7	0.92	112	0.97	321
2nd	9.0	0.17	10.4	0.47	2.2	0.07	74.7	1.5	8.9	0.36	27.6	0.37	4.4	0.10	64.1	2.1	126	0.67	327

3rd	8.7	0.22	10.7	0.33	2.4	0.02	67.3	3.8	7.8	0.41	26.3	1.3	2.7	0.11	62.3	3.2	126	5.6	314
4th	9.1	0.13	10.5	0.41	3.0	0.07	67.4	1.3	7.4	0.60	26.3	0.45	1.9	0.06	64.1	0.90	131	0.81	321
5th	9.1	0.14	10.3	0.34	3.5	0.08	64.2	1.5	7.2	0.23	24.4	0.51	1.6	0.06	60.0	1.5	123	2.3	303
6th	8.8	0.21	11.0	0.22	4.0	0.13	63.0	4.0	7.2	0.27	24.1	1.4	1.6	0.03	59.3	3.3	125	5.7	304
7th	9.4	0.19	10.8	0.37	4.0	0.19	57.5	0.99	6.8	0.17	22.0	0.25	1.5	0.07	53.5	1.3	116	1.2	282
8th	9.4	0.40	11.7	0.42	4.5	0.12	56.1	2.2	7.2	0.19	22.2	0.42	1.7	0.10	54.4	1.6	119	1.9	286
9th	10.5	1.62	12.2	0.58	5.0	0.17	50.5	2.1	7.3	0.53	20.9	0.23	1.8	0.21	54.4	1.1	118	2.8	281
10th	10.1	0.11	12.9	0.13	5.7	0.14	49.5	2.9	7.8	0.24	20.4	0.47	1.9	0.02	54.2	1.0	117	1.9	280

Supplementary Table S5. Significant differences (p-value) obtained between the phenolic compounds of each food EVOO according to the cultivar (Arbequina and Picual) and frying cycle by pairwise comparisons (Wilcox test).

**Anchovies**									
**Frying cycle**	**Hydroxytyrosol**	**Tyrosol**	**Oleocanthalic acid**	**Oleuropein aglycone**	**Oleomissional**	**Ligstroside aglycone**	**Oleokoronal**	**Oleacein**	**Oleocanthal**
1st	*	ns	****	****	***	****	****	*	***
2nd	**	ns	****	****	**	****	****	**	***
3rd	*	*	***	****	****	***	****	**	***
4th	***	ns	****	***	***	***	***	***	***
5th	*	ns	****	***	**	***	****	*	*
6th	****	ns	****	****	**	****	****	****	***
7th	**	ns	****	***	***	****	****	****	****
8th	****	ns	***	****	**	****	****	****	***
9th	**	*	***	***	**	****	****	****	****
10th	***	ns	*	****	ns	**	****	***	**
**Chicken wings**									
**Frying cycle**	**Hydroxytyrosol**	**Tyrosol**	**Oleocanthalic acid**	**Oleuropein aglycone**	**Oleomissional**	**Ligstroside aglycone**	**Oleokoronal**	**Oleacein**	**Oleocanthal**
1st	****	*	**	****	**	****	*	***	****
2nd	ns	ns	**	***	**	****	*	****	***
3rd	***	ns	***	****	****	****	****	****	***
4th	**	ns	****	***	****	***	**	****	****

5th	****	*	****	***	****	****	**	****	****
6th	****	***	****	****	****	****	***	****	****
7th	****	**	****	***	****	****	****	****	****
8th	****	ns	****	**	****	****	****	****	****
9th	***	**	****	***	****	****	****	***	***
10th	***	**	****	**	****	****	***	****	****
**Potatoes**									
**Frying cycle**	**Hydroxytyrosol**	**Tyrosol**	**Oleocanthalic acid**	**Oleuropein aglycone**	**Oleomissional**	**Ligstroside aglycone**	**Oleokoronal**	**Oleacein**	**Oleocanthal**
1st	****	***	*	****	**	****	****	****	***
2nd	****	****	**	****	***	****	**	***	**
3rd	****	****	**	****	***	***	***	**	*
4th	****	***	ns	***	***	****	****	**	**
5th	***	***	**	***	**	***	**	*	ns
6th	****	***	***	***	**	****	***	**	ns
7th	****	****	***	***	***	****	****	***	ns
8th	***	**	***	****	**	****	**	**	ns
9th	****	****	***	****	**	****	****	****	ns
10th	***	***	****	****	****	****	***	***	ns

Fish fingers									
**Frying cycle**	**Hydroxytyrosol**	**Tyrosol**	**Oleocanthalic acid**	**Oleuropein aglycone**	**Oleomissional**	**Ligstroside aglycone**	**Oleokoronal**	**Oleacein**	**Oleocanthal**
1st	***	*	***	***	***	***	****	ns	ns
2nd	*	**	***	****	**	****	**	ns	ns
3rd	**	*	***	**	*	**	**	ns	ns
4th	****	*	*	***	**	**	*	*	***
5th	**	****	****	***	ns	**	ns	**	**
6th	***	**	ns	***	***	***	****	ns	ns
7th	**	***	**	**	**	**	**	ns	ns
8th	***	**	***	****	**	****	**	**	**
9th	**	****	**	***	*	***	***	ns	ns
10th	***	**	***	****	***	****	**	**	**
**Chicken nuggets**									
**Frying cycle**	**Hydroxytyrosol**	**Tyrosol**	**Oleocanthalic acid**	**Oleuropein aglycone**	**Oleomissional**	**Ligstroside aglycone**	**Oleokoronal**	**Oleacein**	**Oleocanthal**
1st	ns	***	*	****	***	****	*	ns	**
2nd	ns	*	**	****	**	****	**	**	****
3rd	ns	***	ns	****	***	****	***	**	***
4th	ns	**	****	****	**	***	**	ns	*
5th	**	**	****	***	**	****	**	**	**
6th	*	****	***	***	**	****	***	*	*

7th	*	**	***	****	**	****	***	*	*
8th	***	****	***	****	****	****	***	***	**
9th	**	**	****	****	**	****	****	***	***
10th	***	**	****	****	**	****	***	**	***
**Eggplants**									
**Frying cycle**	**Hydroxytyrosol**	**Tyrosol**	**Oleocanthalic acid**	**Oleuropein aglycone**	**Oleomissional**	**Ligstroside aglycone**	**Oleokoronal**	**Oleacein**	**Oleocanthal**
1st	**	ns	***	****	***	****	****	**	ns
2nd	***	ns	****	****	**	****	****	**	**
3rd	***	**	****	***	****	****	***	**	*
4th	***	ns	***	****	***	****	***	ns	ns
5th	****	ns	****	****	**	****	***	*	ns
6th	****	**	****	***	**	***	***	ns	*
7th	****	*	***	****	***	****	***	**	ns
8th	*	ns	***	***	**	***	**	*	ns
9th	**	**	***	****	**	****	**	ns	**
10th	****	**	***	***	***	****	****	ns	ns

“****p-value < 0.0001”, “***p-value < 0.0001-0.001”, “**p-value < 0.001-0.01”, “*p-value < 0.01-0.05” and “ns p-value > 0.05”.

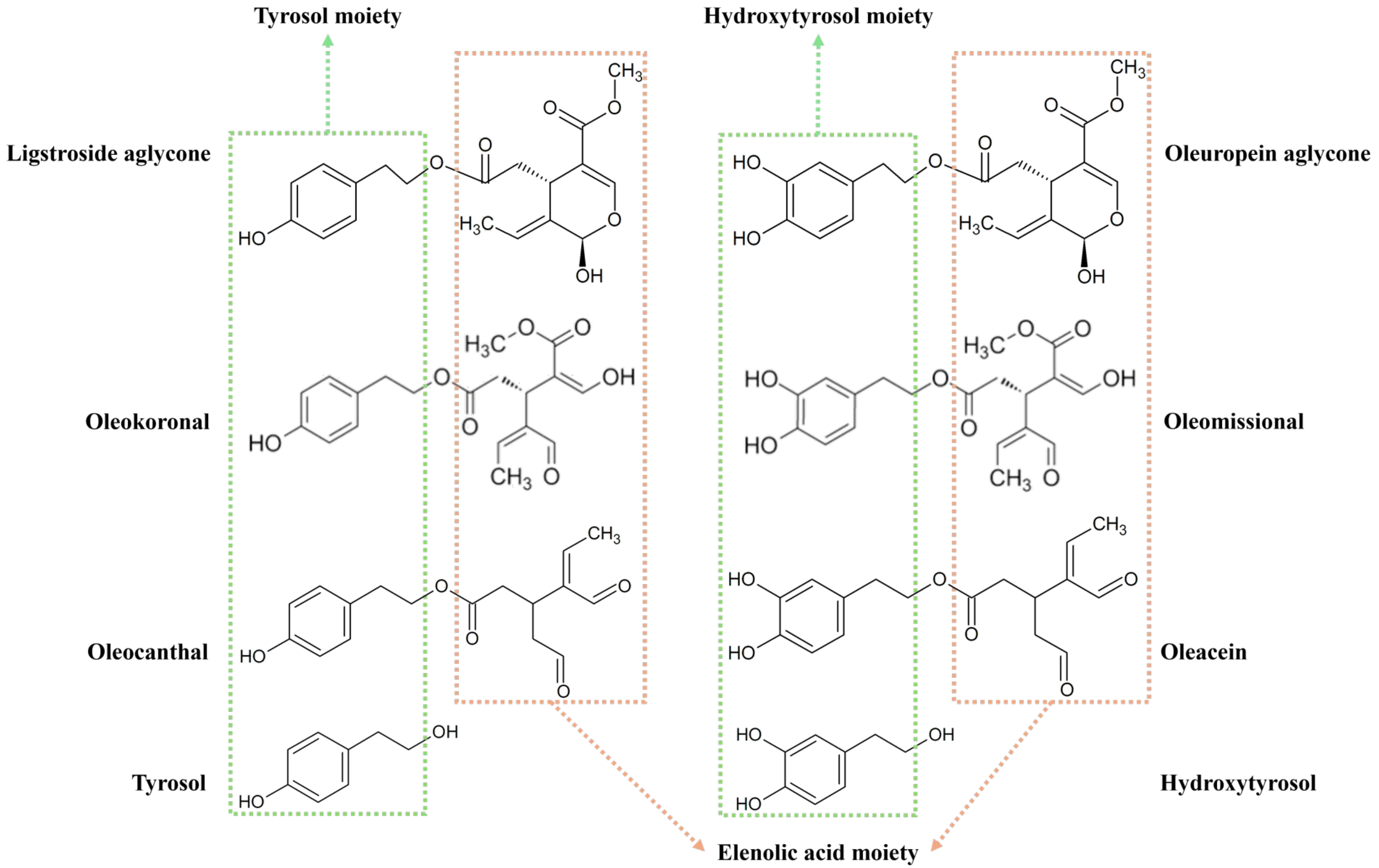

Supplementary Figure S1. Chemical structures of the main secoiridoid derivatives found in olive oil.

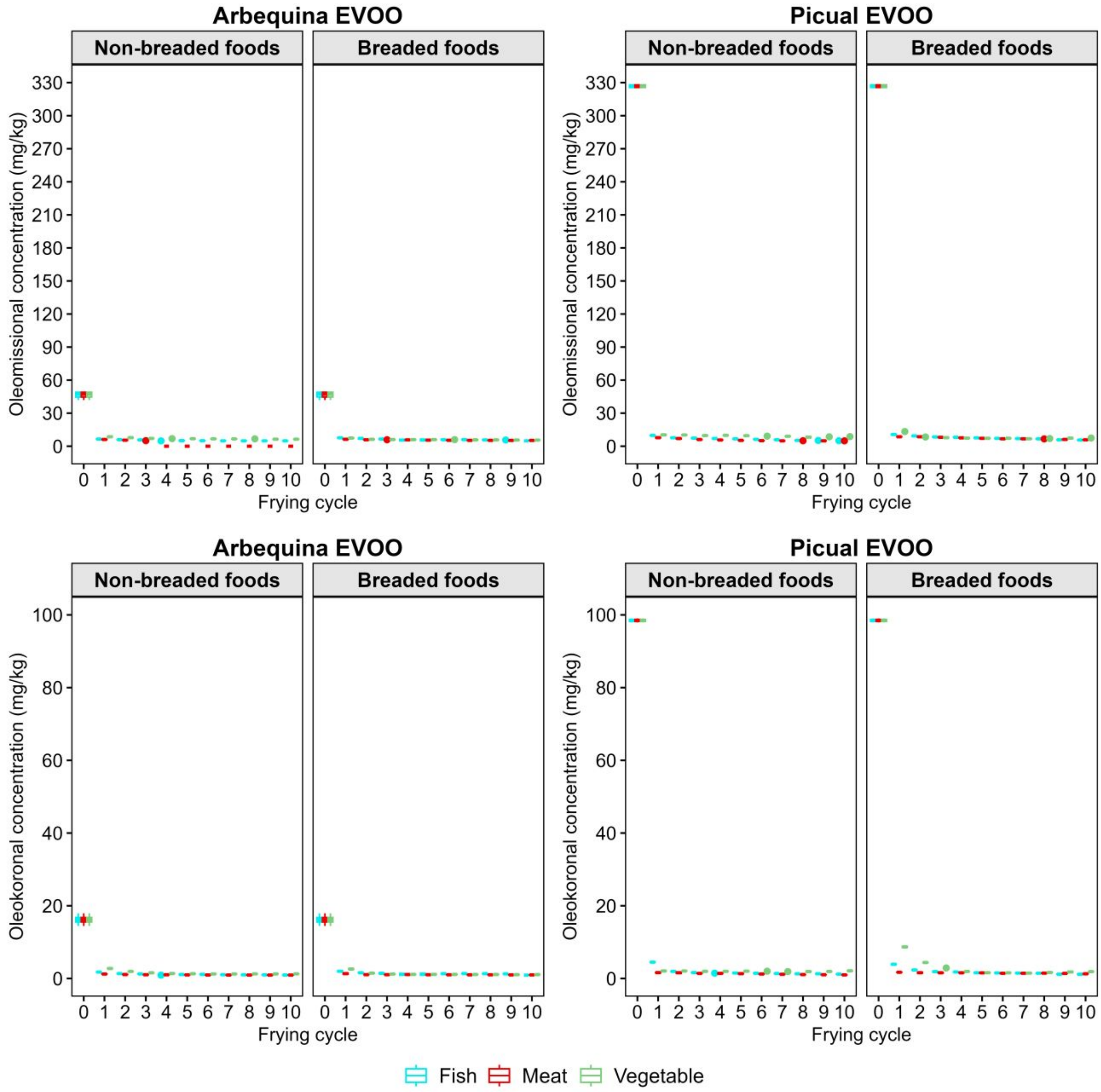

Supplementary Figure S2. Progressive degradation of the oleomissional and oleokoronal in Arbequina and Picual EVOOs during ten frying cycles of non-breaded foods (red, chicken wings; blue, anchovies; green, potatoes) and breaded foods (red, chicken nuggets; blue, fish fingers; green, eggplants). Concentrations were normalized before statistical analysis.
